# Nine *Rhizobium* phage genomes recovered from wastewater in Tempe, AZ, October 2019–March 2020

**DOI:** 10.1128/mra.00680-24

**Published:** 2024-09-19

**Authors:** Brenda Bermudez-Rivera, Brooklyn Hampton, Cian Wheeler, Jacqueline Vargas, Srivatsan Swaminathan, Erin M. Driver, Rolf U. Halden, Arvind Varsani, Matthew Scotch, Temitope O. C. Faleye

**Affiliations:** 1 Biodesign Institute, Center for Environmental Health Engineering, Arizona State University, Tempe, Arizona, USA; 2 School of Life Sciences, Arizona State University, Tempe, Arizona, USA; 3 School of Sustainable Engineering and the Built Environment, Arizona State University, Tempe, Arizona, USA; 4 OneWaterOneHealth, Nonprofit Project of the Arizona State University Foundation, Tempe, Arizona, USA; 5 Biodesign Center for Fundamental and Applied Microbiomics, Center for Evolution and Medicine, School of Life Sciences, Arizona State University, Tempe, Arizona, USA; 6 College of Health Solutions, Arizona State University, Tempe, Arizona, USA; Portland State University, Portland, Orlando, USA

**Keywords:** *Rhizobium* phage, wastewater, Arizona, microvirus, major capsid protein

## Abstract

We describe nine *Rhizobium* microvirus genomes identified in wastewater in Tempe, AZ, USA, between October 2019 and March 2020. The major capsid protein (MCP) encoded in these genomes phylogenetically cluster together and are distinct from the MCPs of *Rhizobium* microviruses identified in Mexico and Argentina.

## ANNOUNCEMENT

*Rhizobium* microviruses (family *Microviridae*) are icosahedral, circular ssDNA (4–6.5 kb) viruses that can be both lytic and lysogenic (1). In November 2020, we detected a *Rhizobium* microvirus in wastewater (WW) in AZ, USA (2). Here, we determine the genomes of nine more *Rhizobium* microvirus from WW in Maricopa County, AZ, USA between October 2019 and March 2020.

For each of the 5 months, 2 L of aggregated (200 mL each) WW collected from 10 sites on the same day was pooled. Each pool was filtered (450 nm), and viruses present in both filtrate and filter-trapped solid (FTS) were concentrated (10 kDa centrifugal filter) to 2 mL each. Specifically, membrane filters with FTS were vortexed at 3,000 rpm for 10 minutes in 50 mL centrifuge tubes containing 25 mL of sterile PCR-grade water and 15 glass beads (3 mm, Cole-Parmer, USA). Afterward, filters were removed, and the mixture was centrifuged for 20 minutes at 3,900 rpm and 4°C. The supernatant was recovered, pooled, and concentrated to 2 mL as above. Subsequently, per month, 150 µL (75 µL each from filtrate and FTS) of concentrate was subjected to nucleic acid extraction (QIAamp Mini Kit), and the extract was used as a template for complete genome (~4.5 kb) amplification as a single contig. Primers Rhph-F (5′-GCCTCGGTTCTGAATTCTGCGGGGTTTACTTCGG-3′) and Rhph-R (5′- TTAAGGCGCGGAGCCTTGGCAACCTTCATTCC-3′) were used alongside Phusion-plus PCR master mix (ThermoFisher Scientific) with reaction conditions 94°C for 3 min, 40 cycles of 94°C for 30 s, 55°C for 30 s, and 68°C for 6 min, and finally 68°C for 10 min (2). Amplicons were resolved on a 1% agarose gel, purified using sparQ magnetic beads (Quantabio, Beverly, MA, USA), subjected to MinION library preparation (SQK-LSK110 and EXP-NBD104), and sequenced on Flongle flow cell R9.4.1. Base calling, trimming, size selection, assembly, and polishing were done using Guppy version 6.2.7 as implemented in MinKNOW version 22.08.4, Porechop version 0.2.4, Geneious Prime version 2023 (3), Flye version 2.9.1, and Medaka version 1.7.2, respectively, as implemented (excluding Geneious Prime) in Nanogalaxy (4). Open reading frames (ORFs) were predicted using GeneMarks version 4.25 (5). Related sequences were downloaded from GenBank. Multiple sequence alignment was done using MAFFT version 7 (6). Pairwise identity analysis was done using SDT version 1.3 (7), and maximum likelihood trees were inferred using IQ-TREE 1.6.12 (8). All software was used with default parameters.

**Fig 1:**
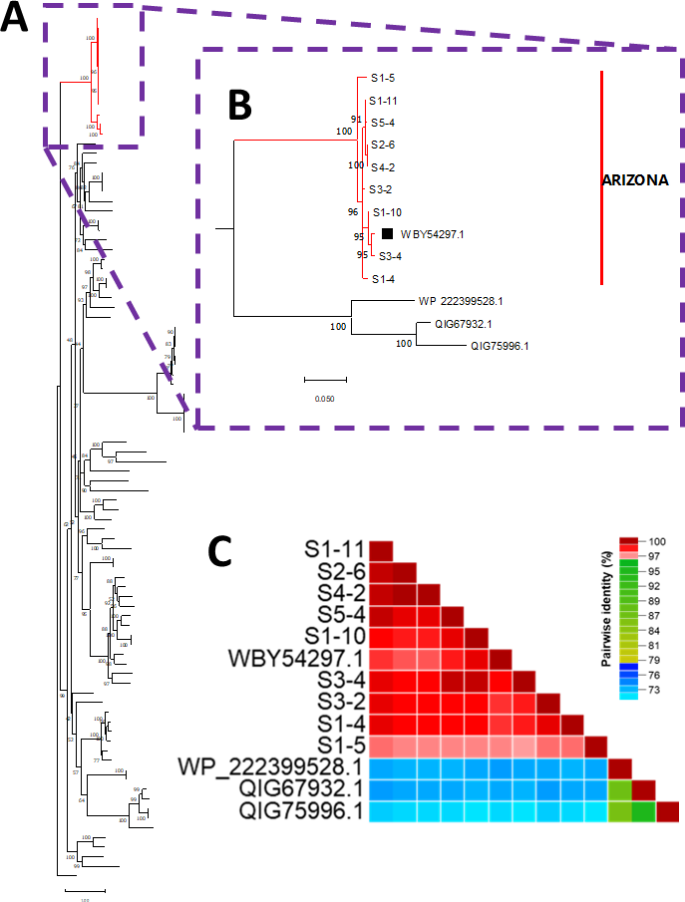
Characterization of *Rhizobium* phages described in this study. (A) A maximum likelihood tree made from *in silico*-translated amino acid sequence of MCPs of *Rhizobium* phages detected in this study and all BLASTp hits in GenBank. The cluster containing *Rhizobium* phages is highlighted in red. (B) An enlarged view of the cluster containing *Rhizobium* phages. Note that phages from Arizona form a distinct cluster, while QIG67932.1 and QIG75996.1 might be representatives of undersampled but unique clusters circulating in Mexico and Argentina, respectively. WP_222399528.1 is a prophage integrated into the genome of a Rhizobacterium. (C) Pairwise identity results of sequences described in panel B. Note that all *Rhizobium* phages in panels B and C have similarity > 70% and those detected in Arizona have similarity > 96%.

The ~4.5-kb amplicons were amplified from each of the five months and sequenced. From 4,983 trimmed reads, we assembled nine contigs (coverage: 91×–1,467×; length: 4,264–4,691 nt; and GC%: 56.5%–57.3%) all of which were most similar (>92%) to OQ184949.1 (Table 1) and encoded the same five ORFs namely, major capsid protein (MCP), endolysin, replication initiation protein, a hypothetical cytochrome and membrane protein, as previously described (2). The MCP of all nine contigs were more than 70% similar to (or less than 30% divergent from) the four *Rhizobium* microviruses previously described and cluster with them in the phylogenetic tree (Fig. 1), confirming all belonged to the subfamily *Amoyvirinae* (1, 2). However, the *Rhizobium* microviruses detected in Arizona WW form a distinct subcluster (Fig. 1).

**TABLE 1:** Properties of the nine *Rhizobium* phage genomes detected in this study^*a*^^,^^*c*^

	^*b*^S1-4	^*b*^S1-5	S1-10	S1-11	S2-6	S3-2	S3-4	S4-2	S5-4
Coverage	91×	127×	370×	467×	505×	156×	305×	1,467×	516×
Length (nt)	4,345	4,264	4,691	4,668	4,688	4,662	4,675	4,669	4,676
GC (%)	57.2	56.5	57	57.3	56.9	56.9	57	56.7	57.2
Query cover	93	94	94	100	100	94	99	100	100
Perc id	93.70	92.69	96.07	98.50	98.74	96.18	98.72	98.59	98.44

^*a*^ Seven (S1-11, S1-10, S2-6, S3-2, S3-4, S4-2, and S5-4) of the genomes encode the complete five ORFs, while one of the ORFs is partial in the remaining two.

^*b*^ Partial ORF 1.

^*c*^ The topmost hit in GenBank to all genomes described here is OQ184949.1. Please note that primers used for the amplification of the complete genome were first described in reference (2). Primer binding sites in the genome are back-to-back and located in the noncoding region between ORF1 and ORF5.

Our results show a unique clade of *Rhizobium* microviruses in WW in Maricopa County, AZ, USA, and suggest the variants detected in Argentina and Mexico might be representatives of undersampled but unique clusters circulating in those regions.

## Data Availability

The reads and genomes described in this study have been deposited in the SRA and GenBank under accession numbers SRR28816390, SRR28816391, SRR28816392, SRR28816393, SRR28816394, and PP826330-PP826338, respectively.
